# Farm Animal Welfare Science in China—A Bibliometric Review of Chinese Literature

**DOI:** 10.3390/ani10030540

**Published:** 2020-03-24

**Authors:** Michelle Sinclair, Yu Zhang, Kris Descovich, Clive J.C. Phillips

**Affiliations:** Centre for Animal Welfare and Ethics, School of Veterinary Science, The University of Queensland, Gatton, QLD 4343, Australia; yu.zhang2014@gmail.com (Y.Z.); k.descovich1@uq.edu.au (K.D.);

**Keywords:** animal welfare, China, science, bibliometric review, livestock production, Asia, pigs, poultry

## Abstract

**Simple Summary:**

For animal welfare scientists and advocates who operate in English, it may be assumed that animal welfare is not an area that receives attention within China; coupled with an awareness that China has not yet opted to enact animal welfare legislation, the reason for this assumption may also be in part due to the perceived lack of animal welfare literature coming from the country. Operating under the hypothesis that animal welfare literature emanating from China may have instead been published in Chinese, rather than English, this study reports the finding of a systematic search of Chinese animal welfare literature on Chinese databases. We searched for articles and research publications released in a recent 10-year period, specifically related to the welfare of the two most commonly farmed land-based animals in China; pigs and chickens, and identified 854 academic publications. In order to facilitate an understanding of Chinese scientific priorities in the field, we further categorised the identified literature into broader approximate categories of welfare freedoms (e.g., freedom from hunger and thirst, freedom to express natural behavior). The identification of a significant animal welfare literature represents an opportunity to increase collaboration with Chinese partners by identifying areas of mutual interest, and to share mutually beneficial knowledge more readily. This could be sustained by increasing the accessibility of Chinese literature to English speakers, and to English literature to Chinese speakers.

**Abstract:**

Farm animal welfare in the People’s Republic of China (henceforth, China) is not well represented in the international scientific literature. This may lead researchers, advocates and those with agricultural partnerships in China to assume that animal welfare is not a field of interest there. This study reports a literature review of published pig and poultry welfare research in China using Chinese scientific databases. We aimed to determine which areas of welfare research have recently received academic attention in China. From an understanding of areas being studied, current and emerging priority areas for research could be determined. This study identified 854 academic publications citing pig or chicken welfare in China published between 2008 and 2018. Within these publications, two broader areas of significant attention were addressed in the context of animal welfare; yield and product quality, such as feeding, biosecurity and antimicrobial resistance, including immunity and second, the relationship of animal welfare with the Chinese philosophy of ‘ecological agriculture.’ Holistic systems were advocated to maximize sustainability and maintain a healthy environment, such as the creation of fermented bedding for pigs. Environmental enrichment was also a focus of attention, demonstrating an interest in animals’ mental welfare, which was usually conjectured from their behavior. Few of the articles were translated into English or other languages and therefore most were largely unavailable to the English-speaking global scientific community. This presents an opportunity to provide relevant animal welfare knowledge, which could improve animal welfare globally. China is a global animal trade leader and the home of the largest agricultural industries in the world. An increase in collaboration on animal welfare research and understanding of the advancements that have been made in China, as reviewed in this manuscript, could advance farm animal welfare from a global perspective.

## 1. Introduction

The People’s Republic of China (henceforth China) has no single legal framework outlining key responsibilities for the welfare and protection of animals [1]. However, since being introduced to mainland China in the 1900s, the concept of ‘animal welfare’ is beginning to be recognized, recently expedited by a growing economy and information accessibility and domestic reform that allows participation in social debate [1]. Before the current growth in prosperity in China [2], animal welfare was generally considered less important than other social issues, such as poverty reduction and improving human welfare. Today it is gaining more attention and generating more discussion in Chinese society [3]. As a similar concept, animal protection ranks amongst the most important social progress movements in China, according to Chinese university students, alongside environmental protection and sustainable development [4]. 

The concept of good welfare for animals is linked to the wider Chinese concept of ‘ecological agriculture’; providing animals with improved health, nutrition and natural conditions that encourage increased productivity, increased profit, as well as being perceived to improve product quality, including taste [5]. Some Chinese livestock companies, such as Guangdong Dexing, Inner Mongolia Yili Industrial Group, Shandong Tuhe Food Co. Ltd and BenLai Group, have responded to the increase in concern for animal welfare by focusing efforts on the supply of products with improved welfare policies [6,7,8,9]. Likewise, one of the largest global food production companies, the Chinese section of CP International, has implemented improved welfare policy and practices in response to increasing consumer concern for animals [10]. ‘Animal welfare’ is also receiving attention from Chinese national governing bodies and authorities, with the establishment of the International Collaborative Committee for Animal Welfare (ICCAW) in 2013. ICCAW is engaged in drafting animal welfare standards and serves as a conduit between international animal welfare non-government organizations, the Chinese livestock community and the central China government [11]. Furthermore, an annual farm animal welfare conference was established in 2016 by the Chinese Government, to promote the improvement of animal welfare in China through the assembly of domestic and international leaders in livestock production, livestock welfare science, animal advocacy and government policy advisors that have interests in animal welfare [3]. 

The concept of animal welfare, as it is understood in Europe, Australia/New Zealand and North America, is still relatively new in China, highlighted by the fact that there is still no clear translation for the term in Mandarin Chinese [12]. In addition, farming practices that have been phased out in other nations for animal welfare reasons remain commonplace in China, such as the use of battery cages for egg-laying chickens [13], sow stalls [14] and teeth trimming of pigs [15]. While approximately 50% of farms in China are still small scale (e.g., fewer than 500 pigs on a single farm), in which the animals are kept in environments that could be considered more natural, the trend is moving towards large scale, intensive farming operations with complex and highly industrialized farming systems as part of a supply chain [16].

Demand for meat is growing worldwide, as a result of population growth and growing affordability of meat in developing country markets [17]. In China, the demand for the main terrestrial animal products (pork, beef, mutton, poultry and eggs) has expanded from 7 kg per capita in 1978 to 25 kg per capita by 2010 [17], with demands forecast to continue increasing exponentially to 55 kg per capita by 2026 [18]. In line with this trend, China’s poultry production has increased most, to an annual output of 19 million tons [19]. To meet this demand further intensification will be required and continued movement towards large scale farms. This has the potential to further jeopardize the welfare of production animals and challenge China’s traditional interest in ‘ecological agriculture.’ Balancing these challenges, while safeguarding animal welfare and economic return, is complex and particularly important because the nation produces more farm animals than any other; 39% of total global production by number [20]. 

Animal welfare in China is best addressed with Chinese solutions, rather than solutions designed by international organizations and dictated by global trends [21]. Nevertheless, the welfare of the animals raised and slaughtered in China is of key interest to trading partners that receive exported produce, particularly those in regions with more stringent animal welfare legislation, practices and expectations [22]. Likewise, the welfare of animals in all countries remains of major importance to those organizations who advocate for animals, including within China. Despite the growing significance of animal welfare within China, the prominence of Chinese agriculture on the global stage and the importance of continued good relationships with international trading partners; little knowledge is available outside of China regarding the state of animal welfare within the nation, and many have a low opinion of Chinese standards [23]. With this lack of knowledge may come an assumption that the state of animal welfare across China has not progressed, and that in some cases cruelty is commonplace. However, good intentions for the welfare of animals have been evident in Chinese literature for centuries. Sixth century literature ‘Qimin Yaoshu’ is one example, and 14th century author Wangzhen Nongshu, specifically cited the need for compassion towards animals [3], albeit both written with regard to the value and productivity of animals for humans. 

In many countries with high welfare standards, reform has been fueled by, and based on, animal welfare science. One example of this can be seen in the findings of the Scientific Veterinary Committee of the European Union, which concluded that serious animal welfare concerns existed for sows in even the best gestation stalls, ultimately resulting in the ban of the system across the European Member States in 2003 [24]. The potentially inaccurate perception that animal welfare is not of interest or concern in China is, at least partially, likely to be influenced by a lack of empirical Chinese animal welfare scientific literature. However, English is the current dominant language of science [25] and commonly used scientific databases are skewed towards English-language journals [26]. This limits the recognition and accessibility of research published in other languages [27]. Relevant studies conducted in China and literature published in Chinese are likely to be infrequently encountered or accessed by the wider scientific community. For this reason, a catalogue of existing animal welfare literature in Chinese and a review of the primary focus of this literature, could serve as a useful tool in better understanding the state of animal welfare in the nation. Furthermore, it could also assist in identifying areas that may benefit from development domestically, with international collaborative support where suitable. It may also serve to identify mutual areas of interest and foster more productive and collaborative relationships; delivering more successful initiatives for improved animal welfare. 

China is a global animal trade leader as well as an important global economic power and the home of the largest animal production industries in the world [20]. Animal agriculture is a particularly important industry for the country, which is likely to continue to increase in importance, alongside crop yields, as the world moves further into anticipated increased food demand [28,29]. It is therefore important that aspects of sustainability, including animal welfare within the pillar of socio-cultural sustainability, are recognized and developed [30,31]. It would be of great utility for China to understand the improved animal welfare practices of international partners and for international partners to understand where the Chinese animal welfare focus has been placed in the past. This study aims to support this endeavor by firstly investigating the reported focus of farm animal welfare science by Chinese scientists, secondly, to quantify the extent of Chinese scientists’ attention to this topic. Lastly, this study aims to identify the extent to which knowledge around animal welfare in China is considered accessible, transferable or shared internationally. To do this, we have conducted a search of chicken and pig scientific literature relevant to animal welfare, created and analysed a library containing this literature.

## 2. Method

### 2.1. Literature Search Strategy 

Literature published between 2008 and 2018 was searched between September and October 2018. This review was conducted by one of the authors (Y.Z.), a Mandarin-speaking Chinese national but also fluent in English and holding a PhD in animal welfare science from an Australian university. The review was focused on chickens and pigs, the two most commonly farmed terrestrial animals in China. The databases used in the search were three Chinese retrieval platforms—VIP Chinese Journal Database (VIP), China National Knowledge Infrastructure (CNKI) and Wanfang Data—and one English database, Web of Science. The following search terms were used—[“猪” or “鸡”] and [“福利” or “康乐”] in the topic for the Chinese databases. The following search terms were used—[“pig” or “swine” or “sow” or “boar,” “piglet” or “poultry” or “chicken” or “broiler” or “hen” or “egg layer” or “egg-layer” or “domestic fowl”] and [“welfare” or “well-being” or “wellbeing”] in the topic for the English database and “China” in the address.

Inclusion criteria used for literature selection were the following—full text articles published in journals or as dissertations; directly related to pig or chicken welfare; Chinese studies or originating internationally but translated to Chinese; affiliations of the first or more authors had to be in China. In total, 505 studies on pig welfare and 349 studies on poultry welfare were identified from the literature search (Table S1). 

### 2.2. Data Extraction and Analysis

Initially two categories were used to classify each item (paper or dissertation). Then each paper or dissertation was assigned to ten primary categories (Table 1). The central primary categories were based on the ‘Five Freedoms’—Freedom from hunger, thirst and malnutrition; Freedom from discomfort and exposure; Freedom from pain, injury and disease; Freedom to express most normal behavior; and Freedom from fear and distress [32,33]. Research items were further categorized by secondary characteristics, treatment factors and measurement variables. Secondary subcategories were created when three or more publications were found to have a similar focus. Subcategories were not mutually exclusive, so a single research item could be included in more than one subcategory. The number of publications in each category was recorded. To further investigate the accessibility of Chinese pig or poultry welfare knowledge, the number of publications available in English and the number of non-Chinese publications translated into Chinese were recorded under each primary category.

To identify which age of animals and production stage received the most scientific focus, this information was extracted from the compiled catalogue. Age of commercial meat chicken, with three fixed age categories and age of commercial egg-laying chicken, with four fixed categories, were based on the definitions by the National Research Council (1994) [34]. Growth phases of pigs reared for meat and production stages of pigs used for breeding were defined according to Food and Drug Administration (2015) [35] and Compassion in World Farming (2019) [36] categories. 

To determine whether the number of publications changed over time, a Pearson’s Correlation test was conducted in R (R Core Team, Vienna, Austria 2009) statistical software in the RStudio interface (RStudio Team, Boston, MA, USA, 2015), with the total number of publications as the dependent variable and year of publication as the independent variable. The final year (2018) was excluded from the analysis as some publications from that year may not have been indexed in databases at the time of data collection. Assumptions for the correlation were checked using Shapiro-Wilk tests for normality of the variables and scatterplot inspection for homoscedasticity and outliers.

## 3. Results

**Figure 1:**
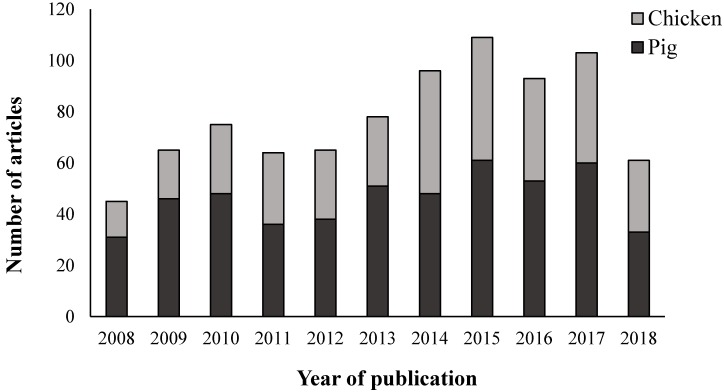
Chinese animal welfare literature published on chickens and pigs from 2008–2018.

In total, 854 articles, published between 2008 and 2018 were identified as relevant to the welfare of pigs (*n* = 505) or chickens (*n* = 349) in the Chinese scientific literature (Figure 1). Over that time period (excluding 2018) there was a significant increase in the number of publications per year (r(8) = 0.90, *p* < 0.001)).

**Figure 2:**
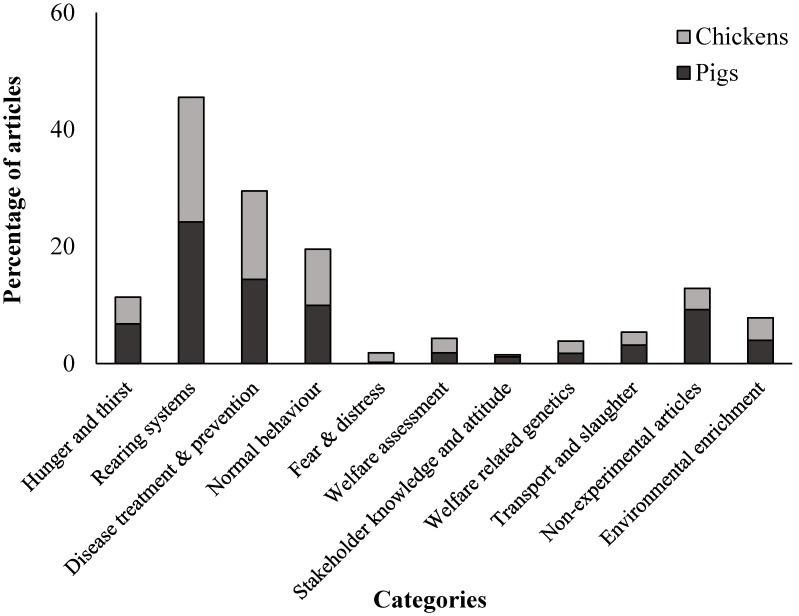
Topic categories of Chinese animal welfare literature on chickens and pigs. The total for each species adds up to more than 100% as categories were not mutually exclusive.

The most common topic categories from the collected dataset were “Rearing systems,” “Disease treatment and prevention” and “Normal behavior” (Figure 2). The least common topics were “Fear and distress” and “Stakeholder knowledge and attitudes.” 

Some similarities and differences in topic occurrence were apparent between the pig and chicken literature (Figure 2, Table 2 and Table 3). Of the pig literature collected, a large percentage was on “integrated management” (30.9%), particularly the secondary sub-category of “rearing methods” (12.7%), as well as “environmental management” (28.1%) and “injury and disease” (15.1%), with secondary subcategories of “immunity” (14.5%)) and “bedding” (9.1%) (Table 2). Behavior was also commonly studied (14.5%), with a predominant focus on general activity such as lying, standing and sitting, but abnormal behavior was also relatively frequently studied. Topics that were not common within the pig literature dataset included “affective states,” “stakeholder attitudes and knowledge” and “genetics”—all comprising less than 2% each of the pig literature collated (Table 2). 

The literature on chickens also had a similar focus on “integrated management” (38.4%), “environmental management” (33.0%) and “rearing” (19.8%) (Table 3). Health-related topics such as “disease and injuries” (28.4%) or their prevention (17.5%) were relatively frequent, with specific focal topics including “feather loss” (10.3%) and “immunity” (13.2%) (Table 3). Behavior was a focus of the chicken literature (22.3%) which included both general behavior as well as abnormal/undesirable behavior such as “aggression” (5.4%) and behavioral indicators of health, for example, “gait score” (11.2%).

In contrast to the pig literature, bedding was infrequently studied (1.2%) in chickens. Chicken-based research on affective states and stakeholder attitudes/knowledge were uncommon (4.0% and 0.9%, respectively). Literature on painful management procedures such as beak trimming was present but uncommon (1.2%) and the related topic of pain relief was absent (Table 3). 

All of the literature published was available in written Chinese, however a subset was also available in another language. In total, 23.3% of pig-focused articles were available in both Chinese and a second language and the same was true for 31.8% of chicken-focused articles relevant. For both species, dual-language items were mostly focused around environmental control, animal discomfort, rearing, pain/injury/disease and normal behavior (Table 4). 

Within species and production systems, there did not appear to be a consistent focus across age/sex classes (Table 5). For example, 40% of all pig articles focused on finishing pigs in meat production, while only 21.0% focused on nursery piglets (Table 5). Articles on pig breeding largely focused on adult females in different stages of the breeding cycle, particularly pregnant (32.5%) and lactating sows (28.9%). For chickens, only 12.3% of the articles focused on breeding animals within the meat industry, with the other age classes having similar levels of representation in this system (starter: 31.0%, grower: 35.2%, finisher: 27.2%). In egg production, the primary focus was on laying hens, with 33.0% of the chicken literature focused on these. Other age classes within egg production comprised less than 15% each of all chicken articles. 

## 4. Discussion

Within this review 854 Chinese animal welfare articles were discovered that had been published in the ten years since 2008. This indicates significant attention to the subject, contrary to the common perception that animal welfare remains an unexplored concept in China. It appears that research aimed at improving conditions and health for pigs and chickens were the most substantial focus in the region.

Within the Chinese animal welfare literature, substantially more focus was placed on pigs than poultry. This could reflect the value of pork industries in international trade or a perceived complexity in providing improved welfare for pigs but may also echo a similar species value system seen in English animal welfare literature. That is, that a pig’s life and intrinsic value is often perceived as more important than that of a chicken, given their closer similarity to humans than chickens and the perception of greater sentience (compared to birds) and therefore increased ability to suffer [37]. 

In general, focus areas in the compiled animal welfare literature tended to be very similar among the species; elements of the rearing environment, which can be related to discomfort, disease and injury and behavioral studies related to natural behaviors. The focus within these general areas, however, differed between the two species. The largest focus; the environment of the rearing system placed substantial attention on bedding systems for pigs, specifically, developing and testing fermented bedding technology. Made of mostly organic material, this substrate was observed to facilitate the decomposition of pig’s excreta and is thought to reduce cleaning time, reduce disease and create a compost that adds richness to agricultural soil; thus increasing sustainability and potentially profit [38]. In terms of the welfare of the pigs, it provides a more comfortable bedding compared to the traditional concrete flooring and opportunities to display natural rooting behavior. Considering that one focus of the Chinese government is on ‘ecological agriculture’ [39], sustainable practices such as this fit within the mandate of improving the environment in general. This focus for animal welfare literature is therefore logical.

While substrate and bedding research in pigs is relevant to welfare because it affects comfort, the issue of discomfort is also being addressed in the poultry research with attention mainly on lighting and temperature control. These factors are also of economic significance as they influence layer hen egg yield and broiler growth rates [40]. Rather than optimization of the farming systems and resources, the focus on these factors is to maximize the animal’s physical productivity, which may at times have positive benefits for welfare (such as more comfortable temperatures) and at other times may be neutral or potentially detrimental to welfare (restricting lighting and forcing molting). 

The second largest focus of the literature on both species was disease and injury. In the pig literature, this was primarily focused on the prevention and control of disease and improving immune responses. This is supported by recent studies, which demonstrate that the global threat antimicrobial resistance poses to animal agriculture and human health alike is taken very seriously in China and could be considered a platform on which to advocate improvements to animal welfare [21,41]. This focus appears to have increased exponentially in China recently, in the wake of an African Swine Fever outbreak that has had a major impact on pig production industries across the country [42]. The focus within the poultry literature was similar; however, controlling the incidence of feather loss, footpad injury and hock injury research was also prioritized, probably motivated by the reduction of carcass and product quality that the injuries can cause, with chickens’ feet being consumed and of value in China [43]. 

The third largest focus within the Chinese literature was on animal behavior. For both species, this primarily addressed feeding and drinking. Related to growth rates, these behaviors are a logical focus when the intention is to increase productivity. The poultry research also included measures of leg health, including walking time and gait scoring. The behavioral literature for pigs included research into lying and standing, as well as social behaviors, such as display of aggressive behaviors, biting and sham chewing. This acknowledgement that pigs are social animals, while still focusing on behaviors that may be problematic for carcass quality [44]. This is echoed by another study in Guangdong province, the home of the largest pork producers in China, in which pig farmers agreed that pigs were intelligent animals, friendly and enjoyed social interaction [45]. 

The areas that received most attention within the compiled Chinese literature indicate a focus on animal welfare tied to improving production, yield, agricultural sustainability and biosecurity, rather than for improving or understanding welfare for the animals’ sake. Livestock leaders in Guangzhou, Beijing and Zhengzhou asked about the most important benefits of addressing animal welfare [5] attested that good welfare improves productivity of the animals, quality of products (including taste) and increased trade opportunities [5]. When the same livestock leaders were asked what they saw as the solutions for improving animal welfare in China they stated that, in addition to creating prescriptive standards, a focus on the business benefits of improving welfare was needed [21]. Considering this, the focus of Chinese animal welfare literature on elements of welfare that result in increased productivity, quality and reduced expense of treatment, indicate that the demonstration of financial benefits from improved animal welfare is of great importance to livestock industries in China.

Welfare topics which received the most attention in the compiled literature offer insight into research priorities in China, however topics that received the little attention offer opportunities for future research and development. One of these areas is pre-slaughter stunning, the pre-slaughter process applied to individual animals to induce unconsciousness and insensibility, so that slaughter can be performed without fear, anxiety, pain, suffering or distress [46]. While limited Chinese animal welfare literature on stunning was discovered in this review (pigs *n* = 8; chickens *n* = 4), it has been identified by Chinese stakeholders as an area of potential development in China. In one survey, Chinese livestock workers ranked the absence of pre-slaughter stunning as the most important farm animal welfare issue in China [47]. In another study, the absence of pre-slaughter stunning was also consistently ranked in a group activity as the most important animal welfare issue in a slaughter context [21]. Furthermore, in a focus group study with livestock leaders, participants consistently suggested that they were ‘extremely’ willing to adopt pre-slaughter stunning, the most willing of stakeholders across the participating Asian nations [48]. In-depth discussion with the same stakeholders suggested that the key ways to increase the uptake of the practice is, firstly, to dispel the perception that stunning negatively impacts meat taste and quality (particularly in south China), secondly, by increasing the accessibility of suitable equipment, and, lastly, providing technical training on usage to operators [48]. As with most animal welfare challenges in China, legislation could be a powerful motivator, however while it is a difficult element of the animal welfare landscape to resolve as long as it remains absent, it does offer a shortcut to motivate uptake of animal welfare practices, including pre-slaughter stunning [49]. Pre-slaughter stunning practices are not used in mainland China at this point, except a few of the major production companies [48]. While the transition in China from smaller companies to major supply chains may resolve this issue, these studies demonstrate an interest and willingness to adopt the practice [21]. Coupled with the demonstrated lack of focus on the issue within Chinese literature to date, there is a substantial opportunity to improve the welfare of animals in China by facilitating a more humane death.

The success of cross-cultural animal welfare initiatives can be enhanced by developing respectful relationships with the most empowered stakeholders and identifying mutually-beneficial outcomes [12]. To that purpose, the findings of this study can be used to ascertain mutual benefits for the Chinese livestock industries and academics, which can be leveraged to progress collaborative relationships that are beneficial and respectful to both parties. The substantial literature presented in this study demonstrates that animal welfare has attracted significant attention in China; however, it may be conceptualized and labelled differently compared to European countries; which should be expected given the vast differences in history, culture and political, social and economic landscapes between the regions. Our study suggests that animal welfare research in China is pragmatic, focused on husbandry practices aimed at good productivity and product quality. The concept of animal welfare for the sake of the animals, for a higher ethical purpose, has not been explored and may therefore not be a fruitful basis for useful animal welfare progression. Opting for language and collaborations in which the net result is improved welfare (and eventually profit), through improving productivity and appealing to Chinese tastes may facilitate more productive partnerships than are otherwise possible. This may mean that successful collaborations to improve animal welfare in China may not focus on animal welfare in the same way that it is approached in key trading partners in the EU, USA and Australia.

Further to this, the difference between Chinese and European approaches to animal welfare may partially explain the potential misconception that China does not value the concept. This misconception may also be partly attributable to the vast cultural differences when approaching how nations treat their progress. Asian culture often values a position that is by nature more quiet, private and modest [50,51], which could at times be contrasted with the cultural approaches to celebrating successes and progress in some of China’s trading partner nations. Chinese research and developments, however, have the potential to reform their livestock industries in future. 

## 5. Applications and Limitations

The primary finding of this study is that there is a substantial body of animal welfare science literature in China, potentially little-known outside of China due to it not being accessible in English, nor being easily accessible to any scientist that does not read the written Chinese language. This finding suggests the existence also of opportunity; to increase knowledge transfer by making key Chinese animal welfare papers available in English, and those from other regions such as Australia/NZ, USA and Europe translated into Chinese. The existence of this literature also suggests the importance for any party developing animal welfare partnerships in China to ensure that they have a thorough understanding of existing literature, in particular its pre-existing foci and conclusions. This study offers a deeper understanding of where attention to farm animal welfare has been directed in China, which assists in the identification of priorities, mutual benefits and opportunities with industry partnerships within China. It also suggests considerable research and development capabilities of Chinese scientists and highlights the areas in which Chinese-led international collaborations could develop science in areas of animal welfare that are being investigated, for example the opportunities and challenges of using China-specific pig or chicken breeds [52]. 

Further research in the form of a comparison of the priorities of European animal welfare science with the findings in this study could be usefully conducted in the future in order to facilitate improved understanding, communication and collaboration across regions.

This literature review focused on the most numerous terrestrial species in Chinese farming—pigs and chickens only. Further investigations through a similar format of literature review could be conducted for other species, such as fish (the most numerous farmed species), dairy cattle, sheep and other frequently consumed aquatic species in China such as the soft-shell turtle; about whom limited animal welfare knowledge is available. In addition, this literature review only covered a 10-year period. As such, this provides a snapshot of welfare science in China during this period but these identified trends are likely to change in the future, particularly in light of the changing livestock landscape due to consumer pressure and disease emergence. This study does, however, provide baseline data to track scientific trends in the future. 

## 6. Conclusions

This study outlines the substantial body of animal welfare science conducted in China and published in Chinese, otherwise unavailable to researchers searching academic databases in English. This study also identifies the research priorities within this work, for both pig and poultry farming. While more focus has been placed on pigs than chickens, the most common area of investigation for both species centers around key environmental factors. In line with the importance of the ‘ecological agriculture’ movement in China, for pigs the literature commonly focused on the use of fermented bedding. For poultry, it was more heavily focused on lighting and temperature control. This was likely to be influenced by motivations to increase productivity and product quality, understood through previous research to be the strongest perceived benefit for improving welfare for livestock stakeholders in China. Reflective of the importance placed on ‘food safety,’ biosecurity and antimicrobial resistance in China, the physical disease burden and use of antibiotics in farm animals was the second most prioritized area of research, coupled with leg breakage injuries for poultry, which impacts carcass quality and the saleability of a body part frequently consumed in the region. The third most common area of animal welfare research was behavior; primarily time spent eating and drinking, which are again direct indicators of productivity and yield. Social and stereotypic behavior of pigs was also a focus, further demonstrating an awareness of the social complexity of pigs as suggested by previous research.

The findings of this study can be used as an opportunity for collaborations based on mutual benefit and respect, with an acknowledgement that animal welfare science does exist in China. While the concept or philosophy motivating the scientific investigation of animal welfare may differ when compared to the contemporary philosophies of other regions, the ultimate outcomes are similar in many contexts and keen interest in industry development is present in China, the world’s most important pig and poultry meat provider.

## Figures and Tables

**Table 1:** Description of categories and subcategories for literature classification.

#	Primary Category of Publication	Subcategory
1	Freedom from hunger, thirst and malnutrition	Feed/diet/nutrition
		Water
2	Freedom from discomfort and exposure	Environment impact and control
		Integrated rearing management
3	Freedom from pain, injury and disease	Pain
		Injury or disease
		Prevention and control
4	Freedom to express normal behavior	Non-experimental article on behavior
		Technology for behavior monitoring or analysis
		Behavior research
5	Freedom from fear and distress	Non-experimental article on psychological welfare
		Measurement of fear responses
6	Welfare assessment & indicators	Welfare assessment
		Welfare indicators
7	Welfare during loading, unloading, transport or slaughter	Non-experimental article on welfare during loading, unloading, transport or slaughter
		Welfare during loading or unloading
		Welfare during transport
		Welfare during slaughter
8	Public or farmers’ knowledge or attitudes towards pig welfare	Knowledge or attitude of pig farmers and farm staff
		Knowledge or attitude of consumers
		Knowledge or attitude of public
9	Non-experimental article on welfare	Non-experimental article on welfare
		Non-experimental article on stress impact
10	Welfare-related genetics and breeding	Genetic improvement and breeding
		Genetic research

**Table 2:** Summary of pig welfare research in Chinese (N = number of publications).

Primary Category	N	Primary Subcategory	N	Secondary Subcategory	N
**1. Freedom from hunger, thirst and malnutrition**	58	Feed/diet/nutrition	53	Functional feeds or ingredients, including feed additives	22
				Feeding strategies	6
				Green feed	6
				Low-protein diet	3
				Mineral nutrition	3
				Nutritional management	3
		Water	5		
**2. Freedom from discomfort and exposure**	241	Integrated management	156	Pig rearing method (incl. use of fermented bedding (45))	64
				Feeding and management of pigs	31
				Monitoring systems	18
				Stocking density or space allowance	10
				Tail docking	8
				International systems	7
				Teeth clipping	6
				Artificial insemination	5
				Castration	5
				Group size	4
				Mixing groups	4
				Weaning piglet management	4
				Pre-weaning piglet management	3
		Environmental management and control	142	Bedding (incl. fermented bedding (45))	46
				Environmental enrichment	34
				Design of pig house and rearing facility	23
				Environmental assessment	18
				Environmental control	12
				Floor type	7
				Temperature, humidity	7
				Ventilation and air quality	9
				Non-experimental article on environmental impact and control	4
				Lighting	3
**3. Freedom from pain, injury and disease**	123	Injury or disease	76	Body injury (incl. skin lesions (22) and tail injury (9))	25
				Lameness, limb-and-hoof disease or injury	17
				Piglet diarrhea	9
				Respiratory or lung disease/damage	8
				Hernia	6
				Incidence rate of diseases	6
				Arthritis	4
				Classical Swine Fever	4
				Gastric diseases	3
				Hemophilus parasuis	3
				Neurological disorder	3
				Non-experimental article on disease	3
				Porcine Reproductive and Respiratory Syndrome	3
		Prevention and control	73	Immunity (incl. indicators of immune response (41))	48
				Specific disease detection technology and treatment	11
				Non-experimental article on causes of diseases, prevention, control	8
				Medicine	6
		Pain	3		
**4. Freedom to express normal behavior**	85	Behavior research	73	Lying	39
				Standing	35
				Sitting	27
				Aggressive behavior	26
				Drinking	25
				Biting behavior (incl. tail (13), ear (5), bar (8), trough biting (2))	23
				Feeding	20
				Elimination	18
				Exploratory behavior	18
				Abnormal behavior (incl. sham chewing (15))	18
				Social behavior (incl. positive and negative social behavior)	11
				Walking	11
				Huddling	10
				Playing	10
				Manipulating behavior (with pen, pen mate, straw, toy)	8
				Resting	6
				Suckling	6
				Sleeping	5
				Vocalizing	5
				Object licking	4
				Nursing behavior	4
				Rooting	4
				Rubbing	4
				Sow posture change	4
				Maternal infanticide	3
				Nest-building	3
				Tongue rolling/playing	3
		Technology for behavior monitoring or analysis	10		
		Non-experimental article on behavior	7		
**5. Freedom from fear and distress**	2	Measurement of fear response	1		
		Non-experimental article on psychological welfare	1		
**6. Welfare assessment & indicators**	16	Welfare indicators	10	Biomarkers	3
		Welfare assessment	7		
**7. Welfare during loading, unloading, transport or slaughter**	27	Welfare during slaughter	13	Slaughter with or without stunning	8
				Pre-slaughter impact and strategy for reducing stress	5
		Welfare during transport	12	Impact of transport stress and strategy for reducing stress	9
				Specific transport stressor	3
		Non-experimental article on welfare during loading, unloading, transport or slaughter	2		
		Welfare during loading or unloading	1		
**8. Public or farmers’ knowledge or attitude on pig welfare**	10	Knowledge or attitude of pig farmers and farm staffs	6		
		Knowledge or attitude of consumers	3		
		Knowledge or attitude of public	1		
**9. Non-experimental article on welfare**	79	Non-experimental article on welfare	59		
		Non-experimental article on welfare related strategy, technology, facility and equipment	18		
		Non-experimental article on stress impact	3		
**10. Welfare related genetic and breeding**	15	Genetic improvement and breeding	6	Stress resistance	3
		Genetic research	9	Genome-wide association study linked to diseases	6

**Table 3:** Summary of chicken welfare research in Chinese (N = number of publications).

Primary Category	N	Primary Subcategory	N	Secondary Subcategory	N
**1. Freedom from hunger, thirst and malnutrition**	39	Feed/diet/nutrition	38	Feed additives	17
				Nutritional management	12
				Feeding strategy and management	7
				Non-experimental article on feed, diet and nutrition	4
		Water	3		
**2. Freedom from discomfort and exposure**	215	Environment impact and control	115	Environmental enrichment	33
				Lighting	24
				Temperature, humidity	23
				Environmental control	15
				Air quality, ventilation and ammonia	15
				Non-experimental article on environmental impact and control	6
				Bedding	4
				Environment assessment	4
		Integrated rearing management	134	Chicken rearing systems	69
				Stocking density	29
				Equipment and technology for rearing management	14
				Rearing management of broilers	8
				Breeding mode	7
				Beak trimming	4
				Rearing management of chicks	4
				Rearing management of layers	3
**3. Freedom from pain, injury and disease**	129	Injury or disease	99	Feather loss	36
				Foot pad injury	35
				Lameness and leg disease or injury	31
				Feather pecking	17
				Feather cleanliness score	13
				Fluctuating asymmetry of legs, wings and tibias	13
				Breast disease	10
				Beak disease or injury	7
				Claw condition including toe damage	6
				Skin wounds	5
				Organ damage	4
				Abnormality of the keel	3
				Ocular health	3
				Skeleton disease or injury	3
		Prevention and control	61	Immunity (incl. indicators of immune response (44))	46
				Non-experimental article on causes of diseases, prevention and control	7
				Specific disease detection technology and treatment	6
				Biosecurity	3
**4. Freedom to express normal behavior**	82	Behavior research	80	Feeding	43
				Gait score	39
				Drinking	38
				Standing	32
				Preening	29
				Walking	27
				Lying	24
				Pecking	23
				Perching	17
				Sand bathing	16
				Foraging	14
				Tonic immobility	14
				Panting	12
				Wing flapping	12
				Stretching	10
				Aggression	19
				Resting	9
				Shaking	9
				Vocalizing	8
				Nesting	7
				Wing lifting	7
				Exploratory activity	6
				Scratching	6
				Head movement	5
				Running	4
				Sitting	4
		Non-experimental article on behavior	2		
**5. Freedom from fear and distress**	14	Measurement of fear response	14		
**6. Welfare assessment and indicators**	21	Welfare indicators	16	Non-experimental article on welfare indicators	13
		Welfare assessment	5		
**7. Welfare during loading, unloading, transport or slaughter**	19	Welfare during slaughter	13	Pre-slaughter impact and strategy for reducing stress	7
				Slaughter with stunning	3
		Welfare during transport	8	Impact of transport stress and strategy for reducing stress	7
				Specific transport stressor	4
		Welfare during loading or unloading	1		
**8. Public or farmers’ attitude**	3	Knowledge or attitude of chicken farmers and farm staffs	2		
		Knowledge or attitude of public	1		
**9. Non-experimental article on welfare**	31	Non-experimental article on welfare	28		
		Non-experimental article on welfare related strategy, technology, facility and equipment	3		
**10. Welfare related genetic and breeding**	18	Genetic improvement and breeding	12	Stress resistance	7
				Non-experimental article on genetic improvement and breeding	3
		Genetic research	6	Genome-wide association study linked to diseases	4

**Table 4:** Transfer and accessibility of animal welfare literature: publications translated from written Chinese into English or from another language into Chinese.

Primary Focus Area—Pigs		Chinese→English	Other Language→Chinese
1	Freedom from hunger, thirst and malnutrition	2	19
2	Freedom from discomfort and exposure	23	16
2a	Environment impact and control	12	4
2b	Integrated rearing management	14	12
3	Freedom from pain, injury and disease	22	14
4	Freedom to express normal behavior	24	10
5	Freedom from fear and distress	1	0
6	Welfare assessment and indicators	4	4
7	Welfare during loading, unloading transport or slaughter	6	4
7a	Welfare during loading or unloading	0	1
7b	Welfare during transport	4	0
7c	Welfare during slaughter	2	3
8	Public or farmers’ attitude	2	3
9	Non-experimental article on welfare	0	4
9a	Non-experimental article on welfare (subcategory)	0	3
9b	Non-experimental article on welfare related strategy, technology, facility and equipment	0	1
10	Welfare related genetics and breeding	8	1
	Total	49	68
**Primary Focus Area—Chickens**		**Chinese→English**	**Other Language→Chinese**
1	Freedom from hunger, thirst and malnutrition	8	11
2	Freedom from discomfort and exposure	52	18
2a	Environment impact and control	36	7
2b	Integrated rearing management	22	12
3	Freedom from pain, injury and disease	41	12
4	Freedom to express normal behavior	21	4
5	Freedom from fear and distress	4	1
6	Welfare assessment and indicators	1	3
7	Welfare during loading, unloading transport or slaughter	3	2
7a	Welfare during loading or unloading	0	1
7b	Welfare during transport	2	1
7c	Welfare during slaughter	2	0
8	Public or farmers’ attitude	2	0
9	Non-experimental article on welfare	0	4
10	Welfare related genetics and breeding	7	5
	Total	69	42

**Table 5:** Animal welfare literature by species, age and production stage (N = number of publications).

Species/System	Age/Stage	Definition	N	%
Chickens: Meat production	For breeding	Breeder males and females	43	12.3
	Starter	0–3 wks old	108	30.9
	Grower	3–6 wks old	123	35.2
	Finisher	6 – > 8 wks old	95	27.2
Chickens: Egg production	For breeding	Breeder males and females	32	9.2
	Starter	0–6 wks old	42	12.0
	Grower	6–12 wks old	37	10.6
	Pre-layer	12–18 wks old	44	12.6
	Layer	> 18 wks old	115	32.9
Pigs: Breeding	Boars	Intact males for breeding/slaughter	121	24.0
	Gilts	Nulliparous females for slaughter/breeding	82	16.2
	Sows	Primiparous or multiparous females	190	37.6
	Gestating sows	Sows that are pregnant	164	32.5
	Farrowing sows	Sows that are farrowing	94	18.6
	Lactating sows	Sows that are producing milk for offspring	146	28.9
	Dry sows	Non-lactating sows that are gestating, awaiting service or barren	54	10.7
Pigs: Meat production	Nursing pigs	Birth until weaning (at ~3–5 weeks old)	165	32.7
	Weaners	Weaning until 10 weeks old	135	26.7
	Nursery pigs	Weaning until end of nursery phase (~18–32 kg)	106	21.0
	Growing pigs	From 18–32 kg until 55–68 kg	137	27.1
	Finishing pigs	From 55–68 kg until market weight for slaughter	202	40.0

## Supplementary Materials

The following are available online at https://www.mdpi.com/2076-2615/10/3/540/s1. Table S1: Catalogue of Chinese scientific literature including farm animal welfare.
